# Diacerein Inhibits Myopia Progression through Lowering Inflammation in Retinal Pigment Epithelial Cell

**DOI:** 10.1155/2021/6660640

**Published:** 2021-07-03

**Authors:** Peng-Tai Tien, Chia-Hung Lin, Chih-Sheng Chen, Ching-Yao Chang, Hsiangyu Ku, Dekang Gan, Yi-Yu Tsai, Jamie Jiin-Yi Chen, Hui-Ju Lin, Lei Wan

**Affiliations:** ^1^Graduate Institute of Clinical Medical Science, College of Medicine, China Medical University, Taichung, Taiwan; ^2^Department of Ophthalmology, China Medical University Hospital, Taichung, Taiwan; ^3^School of Chinese Medicine, College of Chinese Medicine, China Medical University, Taichung, Taiwan; ^4^Division of Chinese Medicine, Asia University Hospital, Taichung, Taiwan; ^5^Department of Medical Laboratory Science and Biotechnology, Asia University, Taichung, Taiwan; ^6^Department of Ophthalmology and Visual Science, Eye and ENT Hospital, Shanghai Medical College, Fudan University, Shanghai 200031, China; ^7^Department of Obstetrics and Gynecology, China Medical University Hospital, Taichung, Taiwan

## Abstract

Myopia is a highly prevalent refractive disorder. We investigated the effect of diacerein on monocular form deprivation (MFD) in hamsters as a possible therapeutic intervention. Diacerein is an anthraquinone derivative drug whose active metabolite is rhein. Diacerein or atropine was applied to the MFD hamsters, and their refractive error and axial length were measured after 21 days. The refractive error (control: −0.91 ± 0.023, atropine: −0.3 ± 0.08, and diacerein: −0.27 ± 0.07 D) and axial length (control: 0.401 ± 0.017, atropine: 0.326 ± 0.017, and diacerein: 0.334 ± 0.016 mm) showed statistically significant differences between control, atropine-treated, and diacerein-treated MFD eyes. Furthermore, we determined the level of transforming growth factor-beta- (TGF-) *β*1, matrix metalloproteinase- (MMP-) 2, type I collagen, interleukin- (IL-) 6, IL-8, and monocyte chemoattractant protein- (MCP-) 1 in the retina. Atropine and diacerein suppressed levels of the myopia-related TGF-*β*1 and MMP-2 while increasing type I collagen expression. They also inhibited the interleukin IL-6, IL-8, and MCP-1 levels. Diacerein reduced the IL-6, IL-8, and MCP-1 expression in ARPE-19 cells. Furthermore, diacerein inhibited inflammation by attenuating the phosphorylation of protein kinase B (AKT) and nuclear factor kappa-light-chain-enhancer of activated B (NF-*κ*B) pathway. This suggests that diacerein has a therapeutic effect on myopia and is a potential treatment option.

## 1. Introduction

Myopia is a common refractive disorder, and its prevalence has been increasing over the past decades [1]. Approximately half of the world population is predicted to be myopic by the year 2050, with as much as 10% being highly myopic [2]. High-degree myopia can lead to an increased risk of cataract, glaucoma, choroidal neovascularization, and macular and retinal complications, which may result in an irreversible vision loss [3, 4]. In addition, patients with myopia may experience progressive changes in eye structure during their lifetime. These changes include loss of scleral tissue, increased type I collagen degradation, and degenerative changes such as atrophy of the retina and choroid [5]. Most myopia results from excessive axial elongation, specifically of the vitreous chamber, which results in increased eye length and blurred vision. Morphological changes in the retinal pigment epithelium (RPE) have been observed in myopia animal models and myopic patients with excessively large eyes [1, 6]. The RPE cells are lost due to chorioretinal atrophy or expanded resulting from passive stretch induced by eye enlargement [1, 6]. The enlarged RPE cells have also been reported in the lid-sutured eyes of a mammalian model [7]. It is thus likely that the RPE play a critical role in eye growth regulation and myopia progression.

Several reports have linked myopia with chronic inflammation [3, 8, 9]. We previously found an increased prevalence of myopia in inflammatory diseases such as type 1 and 2 diabetes, systemic lupus erythematosus (SLE), uveitis, allergic diseases, and Kawasaki disease [3, 8, 9]. The levels of various inflammatory cytokines such as interleukin- (IL-) 6, transforming growth factor-beta- (TGF-) *β*, tumor necrosis factor- (TNF-) *α*, and IL-8 were upregulated in the retina of myopic eyes. In contrast, the anti-inflammatory cytokine, IL-10, decreased in myopic eyes. We also found a decrease in inflammatory transcription factor nuclear factor kappa-light-chin-enhancer of activated B (NF-*κ*B), which is known to induce the expression of matrix metalloproteinase- (MMP-) 2, an important factor in myopia progression [3, 10]. Evidence from animal models of myopia in chicks and tree shrews showed that the MMP-2 expression is upregulated in the sclera [11, 12]. The downregulation of type I collagen was also noted in myopic eyes [3, 9]. The proinflammatory cytokines IL-6, TNF-*α*, and IL-1*β* are primary mediators of chronic inflammation and lead to multiple inflammatory cascades [13]. IL-6, TNF-*α*, and IL-1*β* are also associated with dry eye, uveitis, proliferative diabetic retinopathy, and diabetic macular edema. Overexpression of TNF-*α* and IL-1*β* in the eye initiates a proinflammatory cascade that leads to ocular inflammation [14]. Reports show that high levels of IL-6 and TNF-*α* inflammatory cytokines initiate ocular inflammation and promote myopia progression in hamsters [9]. Other studies have shown that increases in inflammatory cytokines and chemokines play a role in myopia progression [9, 15]. Myopia has become a major public health issue, posing a health and economic burden. Finding an effective drug for the prevention of myopia progression is important. Atropine is currently used to treat myopia and widely used in Asian countries for myopia. In addition, previous research indicated that the expression levels of NF-*κ*B, IL-6, and TNF-*α* were upregulated in myopic eyes and downregulated upon treatment with atropine [3]. However, it has several side effects, such as photophobia and cycloplegia.

Diacerein is an anthraquinone derivative used to treat osteoarthritis, psoriasis, epidermolysis bullosa, type 2 diabetes, and periodontitis [16–18]. Multiple studies have reported that diacerein inhibits the synthesis and activity of proinflammatory cytokines and chemokines such as TNF-*α*, IL-6, IL-1*β*, and monocyte chemoattractant protein 1 (MCP-1) [17, 19, 20]. Numerous clinical studies have proven diacerein as a safe drug with great therapeutic efficacies and minimal side effects [16, 21]. Taken together, we hypothesized that diacerein may prevent myopia progression by inhibiting inflammation.

## 2. Material and Methods

### 2.1. Animals

Three-week-old Golden Syrian hamsters (LASCO, Taiwan) were maintained in a specific pathogen-free animal facility at China Medical University. All procedures were approved by the Institutional Animal Care and Use Committee of China Medical University (approval number: 2017-298-1) and were in accordance with the guidelines for the Use of Animals in Ophthalmic and Vision Research. All animal experiments were performed in the Laboratory Animal Center of China Medical University. The animals were kept under a 12-hour light/12-hour dark cycle. For experiments on hamsters' behavior, the intake of water and food was not limited. We used a previously established hamster model of myopia by MFD with right eyelid fusion for 21 days [3]. The right eyes were sutured with 6-0 PROLENE nonabsorbable suture blue monofilament (W8706, ETHICON, USA) on day 21 after birth. The left eyes were left open and were served as contralateral control eyes. The animals randomly assigned to treatment or control groups (*n* = 10 animals each) received daily applications of drug or phosphate-buffered saline (PBS), respectively, to both eyes. The three groups were (1) control (hamsters received phosphate-buffered saline (PBS)), (2) 1% atropine (Antol Eye Drops 1%, 5 ml/Bot), and (3) 10 mM diacerein (Cat# D9302, Sigma, MO, USA). Eye drops (10 *μ*l) were applied topically to both eyes of the hamsters twice a day (8 AM, 5 PM) until they were euthanized (21 days). All animals were sacrificed in a CO_2_ chamber. Before the animals were anesthetized by CO_2_ gas and sacrificed, the refractive errors and axial lengths of the hamsters were measured. With the animals anesthetized and each eye dilated by a mydriatic (Mydrin-P), the refractive error was measured by using a retinoscopy lens at 50 cm of working distance. The axial length of the eye was defined as the distance from the front of the cornea to the back of the sclera. The axial lengths of each left and right eye were measured by A-scan ultrasonography (PacScan Plus, New Hyde Park, NY, USA). The refractive errors and axial lengths of three independent measurements were averaged.

### 2.2. Immunohistochemistry (IHC)

Eyes were collected from the control, atropine-treated, and diacerein-treated animals, fixed overnight in 4% paraformaldehyde in phosphate buffer, and embedded in paraffin. Eye tissue blocks were sectioned with an 8 *μ*m thickness and mounted on clean glass slides. The slides were exposed to a PBS solution of 5% normal goat serum, blocked for 1 h at room temperature, and then incubated overnight at 4°C with the specific primary antibody IL-6 (1 : 500, Cat# ab6672, Abcam, Cambridge, UK), IL-8 (1 : 200, Cat# MBS551025, MyBioSource, CA, USA), MCP-1 (1 : 500, Cat# ab9669, Abcam, Cambridge, UK), TGF-*β*1 (1 : 100, Cat# ab66043, Abcam, Cambridge, UK), MMP-2 (1 : 500, Cat# ab37150, Abcam, Cambridge, UK), and type I collagen (1 : 100, Cat# GTX20292, GeneTex, Hsinchu, Taiwan). The antigen-antibody link was detected by a secondary antibody tagged with horseradish peroxidase (HRP) for 30 minutes. The color developed by using diaminobenzidine (DAB) working solution (Novolink Polymer Detection System, Leica Biosystems, Newcastle Upon Tyne, UK).

The images of stained sections were taken using an Olympus DP-80 microscope (Olympus Corporation, Center Valley, PA), and quantitative analysis of retina in images was done by using the ImageJ software.

### 2.3. Cell Lines and Cell Culture

The ARPE-19 cells were purchased from the Bioresource Collection and Research Center, Hsinchu, Taiwan (BCRC; BCRC-60, 383). Cells were cultured in Dulbecco's modified Eagle medium (DMEM) (Cat# 12100046, Gibco, Thermo Fisher Scientific, MA, USA) with 10% fetal bovine serum (FBS) (Cat# 16000044, Gibco, Thermo Fisher Scientific, MA, USA) and 1% penicillin (Cat# 15140122, Gibco, Thermo Fisher Scientific, MA, USA), at 37°C in a 5% CO_2_ incubator, replacing the medium every 2-3 days.

### 2.4. ELISA Immunoassay

Cytokines were detected in the supernatants of ARPE-19 cells, seeded at 50,000 cells/well in 24-well plates. ARPE-19 cells were pretreated with 5 ng/ml of different kinds of cytokines (TNF-*α* (Cat# 300-01A, PeproTech, NJ, USA), IL-6 (Cat# 200-06, PeproTech, NJ, USA), IL-1*β* (Cat# 200-01B, PeproTech, NJ, USA), TNF-*α*+IL-6, TNF-*α*+IL-1*β*, IL-6+IL-1*β*, and TNF-*α*+IL-6+IL-1*β*) for 10 mins, then stimulated with diacerein (10 and 100 *μ*M/ml), and incubated for 2 hours. Cell-free supernatants were collected at 2 hours after culture and stored at -80°C until further use. Levels of IL-6, IL-8, and MCP-1 were determined using a human IL-6 (Cat# 88-7066-22, Thermo Fisher Scientific, MA, USA), IL-8 (Cat# 88-8086-22, Thermo Fisher Scientific, MA, USA), and MCP-1 (Cat# 88-7399-22, Thermo Fisher Scientific, MA, USA) ELISA Ready-Set-Go kit following the manufacturer's instructions.

### 2.5. Cell Viability Assay

Cell viability was determined using the MTS/PMS ((3-(4,5-dimethylthiazol-2-yl)-5-(3-carboxymethoxyphenyl)-2(4-sulfophenyl)-2H-tetrazolium, inner salt)/phenazine methosulfate) assay (Cat# G5421, Promega, WI, USA). ARPE-19 cells were seeded in 96-well plates (2 × 10^3^ cells/well). Media containing different concentrations (15.625, 31.25, 62.5, 125, 250, 500, and 1000 *μ*M) of the diacerein were added and incubated for 72 hours. Herein, 20 *μ*l of MTS was subsequently added from a stock solution (2 mg/ml) and incubated for an additional 2 hours. The absorbance was read at 490 nm using the microplate reader 550 model (Bio-Rad).

### 2.6. Western Blot Analysis

ARPE-19 cells were lysed in RIPA (10 mM Tris-Cl, 100 mM NaCl, 1 mM EDTA, 1 mM EGTA, 1 mM NaF, 20 mM Na_4_P_2_O_7_, 2 mM Na_3_VO_4_, 1% Triton X-100, 10% glycerol, 0.1% sodium dodecyl sulfate, and 0.5% deoxycholate) lysis buffer containing protease inhibitors (Roche Applied Science, Madison, USA) and phosphatase inhibitors (Roche Applied Science, USA). Samples (15 *μ*g protein) were loaded on sodium dodecyl sulfate-polyacrylamide gel (SDS-PAGE). The primary antibodies used included AKT (Cat# 9272, Cell Signaling Technology, MA, USA), phosphor-AKT (Ser473) (Cat# 4060, Cell Signaling Technology, MA, USA), NF-*κ*B (Cat# 3034, Cell Signaling Technology, MA, USA), phosphor-NF-*κ*B (p65, Ser536) (Cat# 3031, Cell Signaling Technology, MA, USA), and *β*-actin (Cat# ab8227, Abcam, Cambridge, UK). The primary antibodies were diluted to 1 : 1000 in PBS-5% milk. A goat anti-rabbit IgG conjugated with HRP (1 : 5000 in PBS-5% milk) (Cat# GTX213110-01, GeneTex, CA, USA) was used to detect the protein bands on the polyvinylidene fluoride (PVDF) membrane. Membranes were developed using an enhanced chemiluminescence kit (ECL, Pierce, Thermo Fisher Scientific, MA, USA) and an ImageQuant LAS-4000 Chemiluminescence and Fluorescence Imaging System (GE Healthcare, Illinois, USA).

### 2.7. Software and Statistical Analysis

Each result was expressed as mean ± standard deviation (SD). The unpaired independent *t*-test and one-way ANOVA analysis of variance were performed to compare the differences between the two groups using the GraphPad Prism software. A *p* value < 0.05 was considered to be significant.

## 3. Results

### 3.1. Diacerein Inhibits the Progression of Myopia

**Figure 1:**
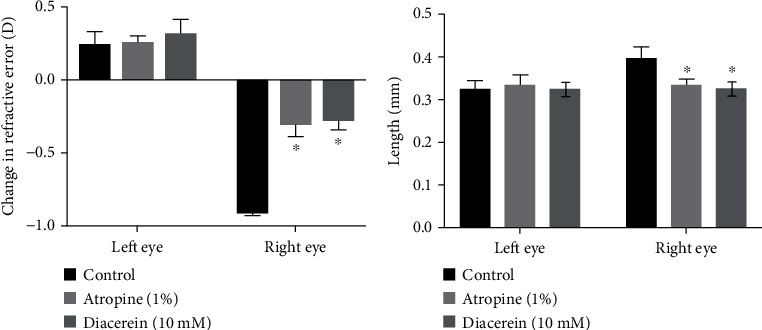
Effect of diacerein on myopia progression. (a) Right eye MFD-induced myopic shift in refraction error was inhibited by atropine (1%) and diacerein (10 mM). The ANOVA test was used to determine significant differences and comparisons between control, atropine (1%), and diacerein (10 mM). The data are expressed as mean ± SD of three independent experiments. ^∗^*p* < 0.05 compared with the control. (b) Right eye MFD-induced axial elongation was inhibited by atropine (1%) and diacerein (10 mM). The ANOVA test was used to determine significant differences and comparisons between control, atropine (1%), and diacerein (10 mM). The data are expressed as mean ± SD of three independent experiments. ^∗^*p* < 0.05 compared with the control.

Atropine (1%) and diacerein (10 mM) were applied to MFD hamsters, and their refractive error and axial lengths were measured 21 days later. MFD induced refractive shift, thus indicating successful myopia development in the control group (right eye) (Figure 1(a)). Hamsters treated with atropine (1%) and diacerein (10 mM) showed a significantly smaller refractive change compared to the MFD group (*p* < 0.05). In addition, changes in the axial length of MFD eyes for the control, atropine-treated, and diacerein-treated MFD hamsters were 0.401 ± 0.017, 0.326 ± 0.017, and 0.334 ± 0.016 mm, respectively (*p* < 0.05; Figure 1(b)). We found no difference in the change in refractive shift and axial length among the contralateral eyes (left eyes). The results indicated that atropine and diacerein treatments significantly inhibit the progression of myopia.

### 3.2. Diacerein Inhibited Myopia Progression through Modulating the Inflammatory Response in the Eyes

**Figure 2:**
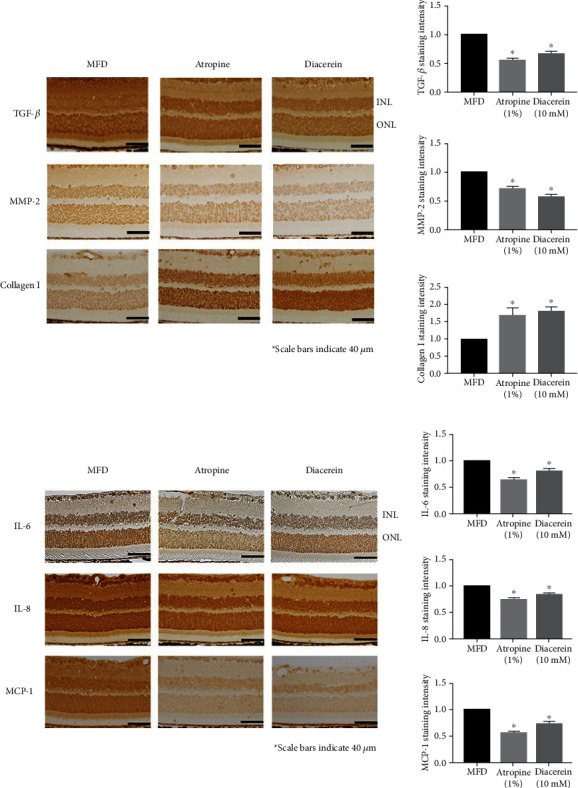
Diacerein affects expression levels of inflammation-related proteins in MFD hamsters. (a) Immunohistochemical analysis of TGF-*β*1, MMP-2, and type I collagen expression in MFD control eyes (right eye MFD), 1% atropine-treated MFD eyes (right eye atropine), and 10 mM diacerein-treated MFD eyes (right eye diacerein). INL: inner nuclear layer; ONL: outer nuclear layer. (b) Immunohistochemical analysis of IL-6, IL-8, and MCP-1 expression in MFD control eyes (right eye MFD), 1% atropine-treated MFD eyes (right eye atropine), and 10 mM diacerein-treated MFD eyes (right eye diacerein). INL: inner nuclear layer; ONL: outer nuclear layer. ANOVA was used for paired comparisons between control, atropine (1%), and diacerein (10 mM). The data are expressed as mean ± SD of three independent experiments. ^∗^*p* < 0.05 compared to the control levels.

Our experimental results showed that the atropine and diacerein treatment resulted in a significant (*p* < 0.05) attenuation of TGF-*β*1 and MMP-2 and an increase in type I collagen levels compared to the control group (right eye MFD) (Figure 2(a)) in the retina of myopic eyes. The results suggest that we successfully induced myopia in experimental animals.

Diacerein and atropine treatment resulted in a significant (*p* < 0.05) attenuation of IL-6, IL-8, and MCP-1 levels in myopic eyes compared to the control group (right eye MFD) (Figure 2(b)). The results suggested that diacerein had similar regulatory effects as atropine and that it inhibited myopia progression in MFD eyes by modulating the changes in the tissue remodeling proteins and the inflammatory effects.

### 3.3. Diacerein Reduced the Inflammatory Cytokine Production in ARPE-19 Cells

The immunofluorescence result showed increased TNF-*α* expression levels of RPE in the MFD eye. The diacerein treatment resulted in attenuation of TNF-*α* levels in the myopic (right) eyes compared to the control group (right eye MFD) (Supplementary Figure 1). Therefore, we used human retinal pigment epithelial cells, ARPE-19, to study the molecular mechanisms on how diacerein lowers the ocular inflammation.

**Figure 3:**
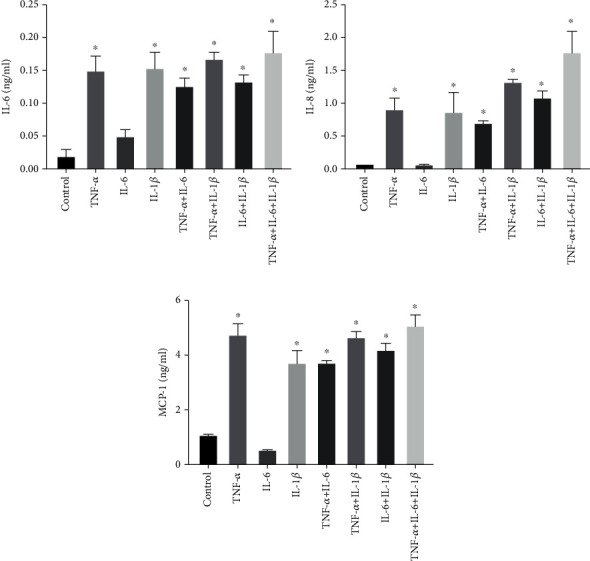
Cytokine-induced proinflammatory gene expression in ARPE-19 cells. The levels of (a) IL-6, (b) IL-8, and (c) MCP-1 in ARPE-19 cells were measured using the ELISA Ready-Set-Go kit. TNF-*α*+IL-6+IL-1*β* treatment significantly increased the levels of IL-6, IL-8, and MCP-1. The data are expressed as mean ± SD of three independent experiments. ^∗^*p* < 0.05 compared with the control level.

The effect of treatment of ARPE-19 with diacerein on proinflammatory cytokine expression levels was investigated. ARPE-19 cells were treated with 5 ng/ml of different cytokines as well as combinations of cytokines (TNF-*α*, IL-6, IL-1*β*, TNF-*α*+IL-6, TNF-*α*+IL-1*β*, IL-6+IL-1*β*, and TNF-*α*+IL-6+IL-1*β*) for 10 min. The cytokines were then removed, and the cells were incubated in fresh media for 2 hours. ELISA analysis demonstrated a significant increase in the levels of IL-6, IL-8, and MCP-1, detected by the different cytokine treatments in ARPE-19 cells (Figures 3(a)–3(c)). TNF-*α* and IL-1*β* significantly increased the levels of IL-6, IL-8, and MCP-1. The levels were further increased when combining treatment with TNF-*α* and IL-1*β*. The IL-6 exhibited less effect on inflammatory cytokine production. However, when combined with TNF-*α*, IL-6, and IL-1*β*, it showed the highest level of inflammatory cytokine production. A synergistic effect was found among TNF-*α*, IL-6, and IL-1*β*.

**Figure 4:**
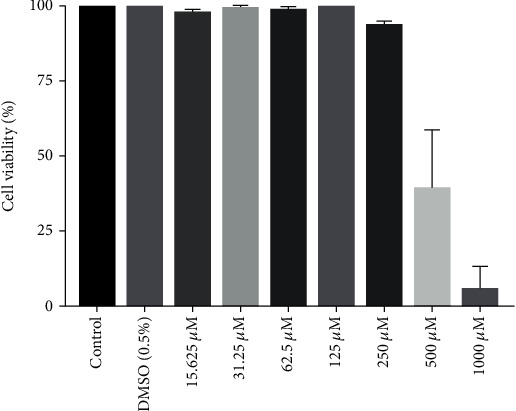
Viability of ARPE-19 cells treated with different concentrations of diacerein. Cell viability was determined by the MTS assay. Results show that diacerein had no significant cytotoxic effect on ARPE-19 cells. The data are expressed as mean ± SD of three independent experiments.

**Figure 5:**
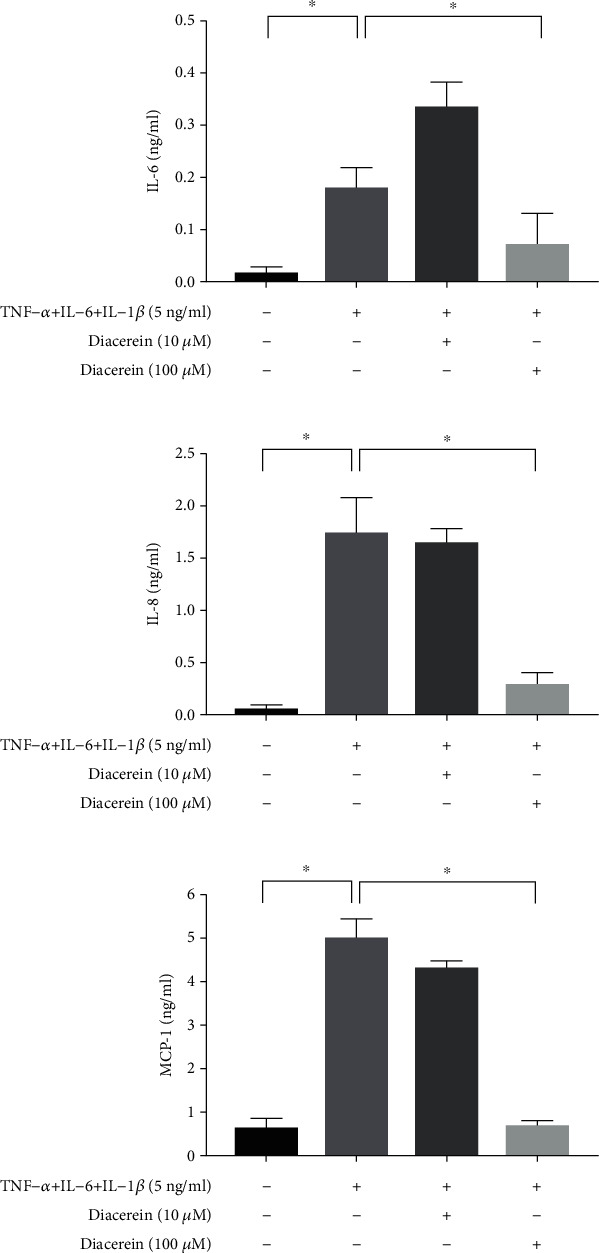
Diacerein protects against cytokine-induced inflammation in ARPE-19 cells. The levels of (a) IL-6, (b) IL-8, and (c) MCP-1 in ARPE-19 cells were measured using the ELISA Ready-Set-Go kit. Diacerein (100 *μ*M) significantly suppressed the expression of IL-6, IL-8, and MCP-1 induced by TNF-*α*+IL-6+IL-1*β*. The data are expressed as mean ± SD of three independent experiments. ^∗^*p* < 0.05 compared with the control level.

We then assessed whether diacerein would inhibit the inflammatory reactions induced by TNF-*α*+IL-6+IL-1*β* treatment in ARPE-19 cells. The MTS assay was used to evaluate the cytotoxic effect of the 15.625 to 1000 *μ*M diacerein for 72 hours (Figure 4). No significant cytotoxic effect was changed on ARPE-19 cells treated with diacerein at concentrations ranging from 15.625 to 125 *μ*M. According to the MTS assay results, diacerein concentrations of 10 and 100 *μ*M were chosen for the following experiments. Diacerein (100 *μ*M) significantly suppressed the expression of IL-6, IL-8, and MCP-1 induced by TNF-*α*+IL-6+IL-1*β* (Figures 5(a)–5(c)). However, 10 *μ*M diacerein did not inhibit the expression of IL-6, IL-8, and MCP-1 induced by TNF-*α*+IL-6+IL-1*β*.

### 3.4. Diacerein Attenuates the Activation of AKT and NF-*κ*B Signaling Pathways in ARPE-19 Cells

**Figure 6:**
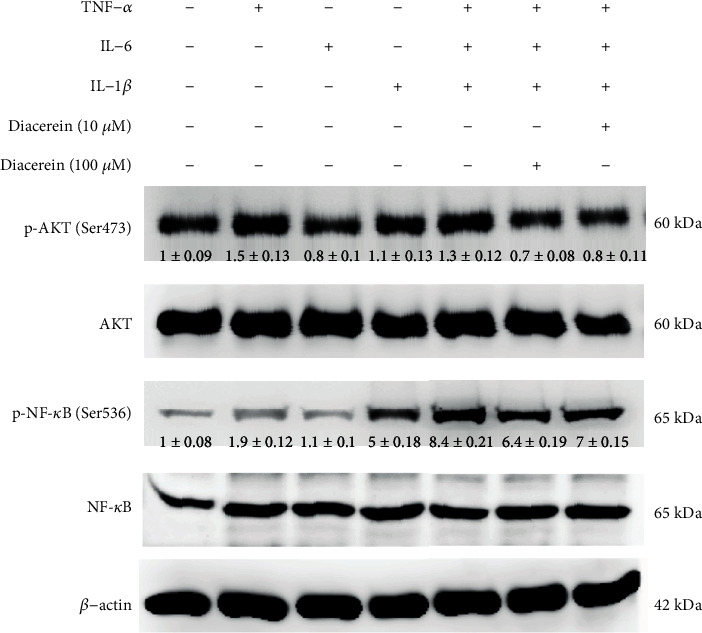
Diacerein inhibits cytokine-induced inflammation through attenuating AKT and NF-*κ*B phosphorylation in ARPE-19 cells. TNF-*α*, IL-6, and IL-1*β* facilitated an increase in AKT and NF-*κ*B phosphorylation levels in treated ARPE-19 cells compared to control cells. The phosphorylation level of AKT and NF-*κ*B determined by western blotting. *β*-Actin was used as a reference control. Full-length blots are presented in Supplementary Figure 2. Quantification of protein expression levels by normalization to the internal control *β*-actin. Depicted western blots are one representative figure of three independent experiments. Results are the mean ± SD of three independent experiments.

Next, we aimed to elucidate the molecular mechanism by which diacerein regulates inflammation. ARPE-19 cells were treated with TNF-*α*, IL-6, IL-1*β*, or TNF-*α*+IL-6+IL-1*β* for 10 min. Treatment media were subsequently removed, and fresh media with or without diacerein (10 or 100 *μ*M) were applied and incubated for 2 hours. Our results showed that TNF-*α*+IL-6+IL-1*β* caused a substantial increase in phosphorylation of AKT and NF-*κ*B in the ARPE-19 cells (Figure 6). Quantitative analysis of the bands showed that diacerein (10 and 100 *μ*M) treatments attenuated the TNF-*α*+IL-6+IL-1*β*-induced phosphorylation of AKT and NF-*κ*B. This result indicates that diacerein inhibits inflammation through a downregulation of the AKT and NF-*κ*B pathways.

## 4. Discussion

Ocular inflammation has been indicated to be involved in the pathophysiology of various retinal diseases, including myopia, AMD, and uveitis [22]. TNF-*α* and IL-6 are proinflammatory cytokines and play a major role in retinal inflammation [23]. Previous studies have also shown that inflammatory mediators, including IL-6, TNF-*α*, TGF-*β*, IL-1*β*, NF-*κ*B, and MMP-2, were upregulated in an MFD animal model [3]. Similar results were observed by lipopolysaccharide (LPS) treatment in the ARPE-19 and RPE cells [3]. In addition, previous research showed that myopia progression was slowed down by cyclosporine A (CSA) and CSA treatment further reduced the expression of IL-6, TNF-*α*, c-FOS, and NF-*κ*B in the eye [9]. Therefore, one can assume that inflammation plays a vital role in myopia progression. Diacerein is an anti-inflammatory drug that is an inhibitor of proinflammatory IL-1*β* [24–27]. Diacerein may have therapeutic applications to attenuate cytokine-induced development of myopia progression. To confirm the effect of diacerein on myopia progression, diacerein (10 mM) and atropine (1%) were applied to MFD hamsters and their refractive error and axial length were measured 21 days later. The experimental findings in hamsters corroborate our hypothesis that diacerein is a potential inhibitor of myopia progression. Thus, there is increasing experimental and clinical evidence that inflammation may potentially influence the development of myopia. The present study provides the first evidence that diacerein suppressed protein expression of TGF-*β*1, MMP-2, type I collagen, IL-6, IL-8, and TNF-*α* in MFD hamsters.

The inflammatory cytokines TNF-*α*, IL-6, and IL-1*β* are known to have a predominant role in inflammatory-related disease. Several cell types, including RPE, vascular cells, endothelial cells, fibroblasts, and astrocytes, increase IL-6 expression when exposed to TNF-*α* and IL-1*β* [28, 29]. In addition, not only does the IL-1*β* mediate inflammation at the tissue level but also it propagates inflammation by inducing other proinflammatory cytokines, such as TNF-*α* and IL-6 [23]. The effects of IL-6 in uveitis may be further augmented by ambient intraocular TNF-*α* and IL-1*β* [30]. Any abnormalities in IL-6, TNF-*α*, and IL-1*β* cytokine signaling may induce several disorders [31]. Numerous reports demonstrate that TNF-*α* is a major NF-*κ*B dependent inflammatory cytokine, which results in increased risk of ocular inflammatory disease, including uveitis and age-related macular degeneration [32]. The eye may be a target of inflammatory attack due to overactivity of TNF-*α* and NF-*κ*B. Recent reports show that anti-TNF-*α* and anti-NF-*κ*B drugs could prevent ocular inflammation, especially uveitis [33]. Previously, we and others have found that inflammatory cytokines such as IL-6, IL-8, and MCP-1 are overexpressed in myopic eyes and associated with myopia progression [3, 34]. Chemokine production by RPE is regulated by proinflammatory cytokines, of which TNF-*α* and IL-1*β* significantly increased the production of IL-6, IL-8, and MCP-1. These are the most common cytokines produced by RPE cells, reacting to different inflammatory stimuli and known to play a key role in retinal inflammatory diseases [35, 36]. In the present study, stimulation of ARPE-19 cells with 5 ng/ml of different cytokines and in combination (TNF-*α*+IL-6, TNF-*α*+IL-1*β*, IL-6+IL-1*β*, and TNF-*α*+IL-6+IL-1*β*) induced an increased production of IL-6, IL-8, and MCP-1. The combination of cytokines (TNF-*α*+IL-6+IL-1*β*) had the highest induced effect on inflammation. The release of inflammatory cytokines leads to a further release of more inflammatory factors that cause inflammation-related diseases [37, 38]. Similar to the findings of previous studies, treatment of corneal epithelial (CEP) cells with TNF-*α*+IL-6 exhibited even higher expression levels of TNF-*α*, IL-6, and IL-8 [9]. Thus, a vicious cycle develops between continuously induced TNF-*α*, IL-6, and IL-1*β* inflammatory responses in the RPE cells that would affect the levels of IL-6, IL-8, and MCP-1 intraocularly, which would promote myopia progression.

In this study, we show that diacerein reduced the combination of cytokines (TNF-*α*+IL-6+IL-1*β*) which induced increases in IL-6, IL-8, and MCP-1 expression in ARPE-19 cells. As inflammation may play a key role in myopia progression, the cell signaling pathway involved in the release of cytokines and chemokines in RPE cells is of importance. *In vitro* studies demonstrate that cytokine (TNF-*α*+IL-6+IL-1*β*) activation of AKT and NF-*κ*B can further affect inflammation. Thus, inhibition of AKT and NF-*κ*B is important for controlling the inflammatory conditions. Diacerein has been confirmed to inhibit inflammatory effects *via* inhibiting the activation of AKT and NF-*κ*B signaling pathways [39, 40]. The results of the present study show that 2 hours after the diacerein treatment, phosphorylation levels of AKT and NF-*κ*B are decreased markedly compared to those of cells treated with cytokines. The study on animal eyes is quite similar to one conducted in the ARPE-19 cells. Upon treatment with atropine and diacerein, the myopia progression was inhibited. Furthermore, consistent with our cell data, IL-8 and MCP-1 levels were elevated in myopic eyes compared to nonmyopic eyes. Treatment with atropine and diacerein resulted in a decreased expression of IL-8 and MCP-1 staining compared to the control group. TGF-*β* is a pleiotropic cytokine with several different roles in human inflammation disease and disorders, including myopia. TGF-*β* is also known to affect expression of the MMP family, including MMP-2, in human eyes [41, 42]. The expression of MMP-2 is an important promoter of tissue remodeling in the eye. TGF-*β* also induces or increases the expressions of IL-8 and IL-1*β* during the modulation of the inflammatory response. In high myopic eyes, increased expression levels of TGF-*β* and MMP-2 and decreased type I collagen resulted in scleral thinning, the major structural protein in the sclera [43]. In this study, the MFD hamsters expressed higher TGF-*β*1 and MMP-2 and lower type I collagen. We show that atropine (1%) and diacerein (10 mM) suppress the levels of TGF-*β*1 and MMP-2 and increase type I collagen expression in MFD hamsters.

Although we found an inhibitory effect of diacerein in slowing myopia progression through lowering the retina inflammation, we could not explain how ocular surface diacerein application would modulate retina inflammation. To determine the intraocular diacerein concentration is important in understanding the effect of diacerein on retina and is also important information to design a suitable eye drop formulation for clinical studies. The dose of diacerein causing adverse effects and safety range of its dose should also be determined before clinical trials. Moreover, the efficacy of diacerein in lens-induced myopia should be evaluated.

## 5. Conclusions

This study shows that the proinflammatory cytokine combinations of TNF-*α*+IL-6+IL-1*β* significantly increase the expression of IL-6, IL-8, and MCP-1 in ARPE-19 cells. Diacerein also decreases cytokine-induced inflammation through inhibiting the AKT and NF-*κ*B pathways. In addition, we have shown that the diacerein could reduce the expression of TGF-*β*1 and continue to inactivate the expression of MMP-2. These effects subsequently reduce increases in type I collagen degradation, which preserves collagen expression at levels that sustain tissue structural integrity. To conclude, diacerein is a new potential strategy for the management of myopia, and its continued usage and research will allow for a new class of treatment for myopia.

## Data Availability

The data used to support the findings of this study are available from the corresponding author upon request.

## Abbreviations

MFD: Monocular form deprivation
TGF-*β*: Transforming growth factor-beta
MMP-2: Matrix metalloproteinase
IL: Interleukin
MCP-1: Monocyte chemoattractant protein-1
AKT: Protein kinase B
NF-*κ*B: Nuclear factor kappa-light-chin-enhancer of activated B
TNF: Tumor necrosis factor
SLE: Systemic lupus erythematosus
Rhein: 4,5-Dihydroxy-9,10-dioxo-9,10-dihydroanthracene-2-carboxylic acid
PBS: Phosphate-buffered saline
IHC: Immunohistochemistry
DMEM: Dulbecco's modified Eagle medium
FBS: Fetal bovine serum
MTS: 3-(4,5-Dimethylthiazol-2-yl)-5-(3-carboxymethoxyphenyl)-2(4-sulfophenyl)-2H-tetrazolium, inner salt
PMS: Phenazine methosulfate
SDS-PAGE: Sodium dodecyl sulfate-polyacrylamide gel
RPE: Retinal pigment epithelium
LPS: Lipopolysaccharide
CEP: Corneal epithelial cell.

## Supplementary Materials

Supplementary Materials Supplementary Figure 1: increased expression of TNF-*α* in the retina of hamsters after MFD-induced myopia progression. Supplementary Figure 2: original blots of Figure 6. Supplementary Figure 3: immunohistochemical analysis of TGF-*β*1, MMP-2, type I collagen, IL-6, IL-8, and MCP-1 expression in right eye control of a normal hamster. Supplementary Figure 4: immunohistochemical and analysis of TGF-*β*1, MMP-2, type I collagen, IL-6, IL-8, and MCP-1 expression in left eye control, 1% atropine, and 10 mM diacerein of a hamster.
