# Supplementary material for: Diacerein Inhibits Myopia Progression through Lowering Inflammation in Retinal Pigment Epithelial Cell
Source: Mediators Inflamm. 2021 Jul 3;2021:6660640. doi: 10.1155/2021/6660640 (PMC8275387; doi:10.1155/2021/6660640)
Supplement: Supplementary Materials — Supplementary Figure 1: increased expression of TNF-α in the retina of hamsters after MFD-induced myopia progression. Supplementary Figure 2: original blots of Figure 6. Supplementary Figure 3: immunohistochemical analysis of TGF-β1, MMP-2, type I collagen, IL-6, IL-8, and MCP-1 expression in right eye control of a normal hamster. Supplementary Figure 4: immunohistochemical and analysis of TGF-β1, MMP-2, type I collagen, IL-6, IL-8, and MCP-1 expression in left eye control, 1% atropine, and 10 mM diacerein of a hamster. [file 6660640.f1.docx]

**Diacerein inhibits myopia progression through lowering inflammation in retinal pigment epithelial cell**

Peng-Tai Tien^a,b,&^, Chia-Hung Lin^c,&^, Chih-Sheng Chen^c,d^, Ching-Yao Chang^e^, Hsiangyu Ku^f^, Dekang Gan^f^, Yi-Yu Tsai^a,b^, Jamie Jinn-Yi Chen^b^, Hui-Ju Lin^b,c^, Lei Wan^c,e,g^

^a^Graduate Institute of Clinical Medical Science, College of Medicine, China Medical University, Taichung, Taiwan

^b^Department of Ophthalmology, China Medical University Hospital, Taichung, Taiwan

^c^School of Chinese Medicine, College of Chinese Medicine, China Medical University, Taichung, Taiwan

^d^Division of Chinese Medicine, Asia University Hospital, Taichung, Taiwan

^e^Department of Biotechnology, Asia University, Taichung, Taiwan

^f^ Department of Ophthalmology and Visual Science, Eye and ENT Hospital, Shanghai Medical College, Fudan University, Shanghai 200031, People’s Republic of China

^g^Department of Obstetrics and Gynecology, China Medical University Hospital, Taichung, Taiwan

**Supplementary Figure 1:**

**
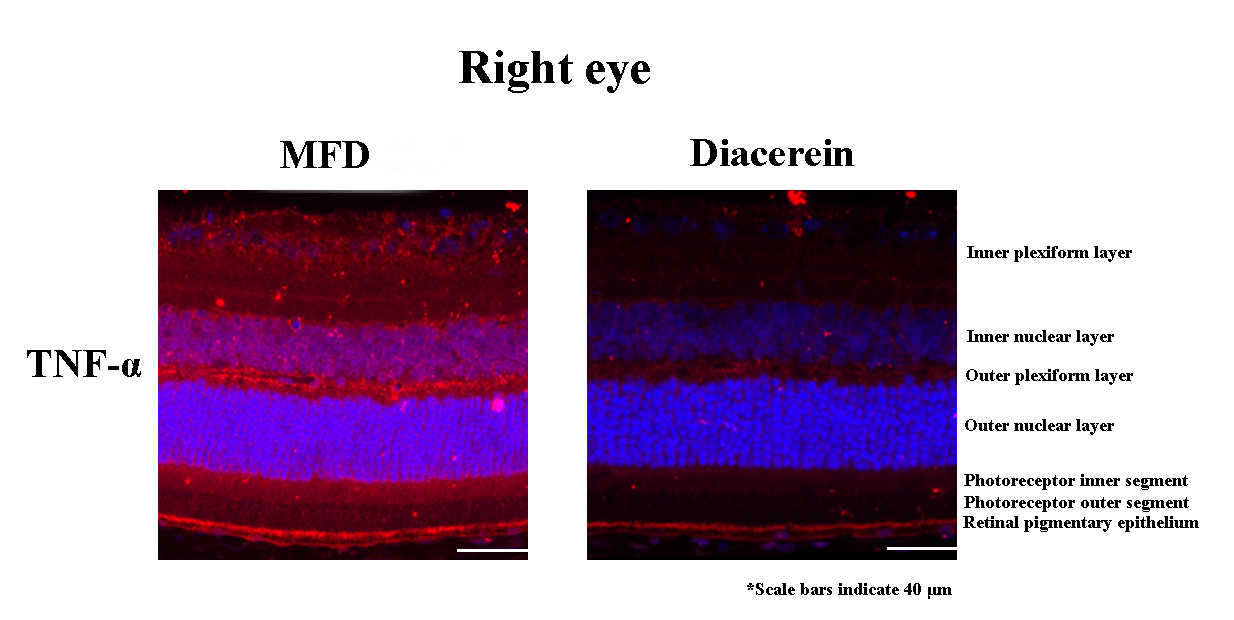
**

**Supplementary Figure 1: Increased expression of TNF-α in retina of hamsters after MFD induced myopia progression**

Immunofluorescence analysis of TNF-α expression in MFD eyes (Right eye MFD), and 10 mM diacerein-treated MFD eyes (Right eye diacerein).

**Supplementary Figure 2: Original Blots of Figure 6**

**
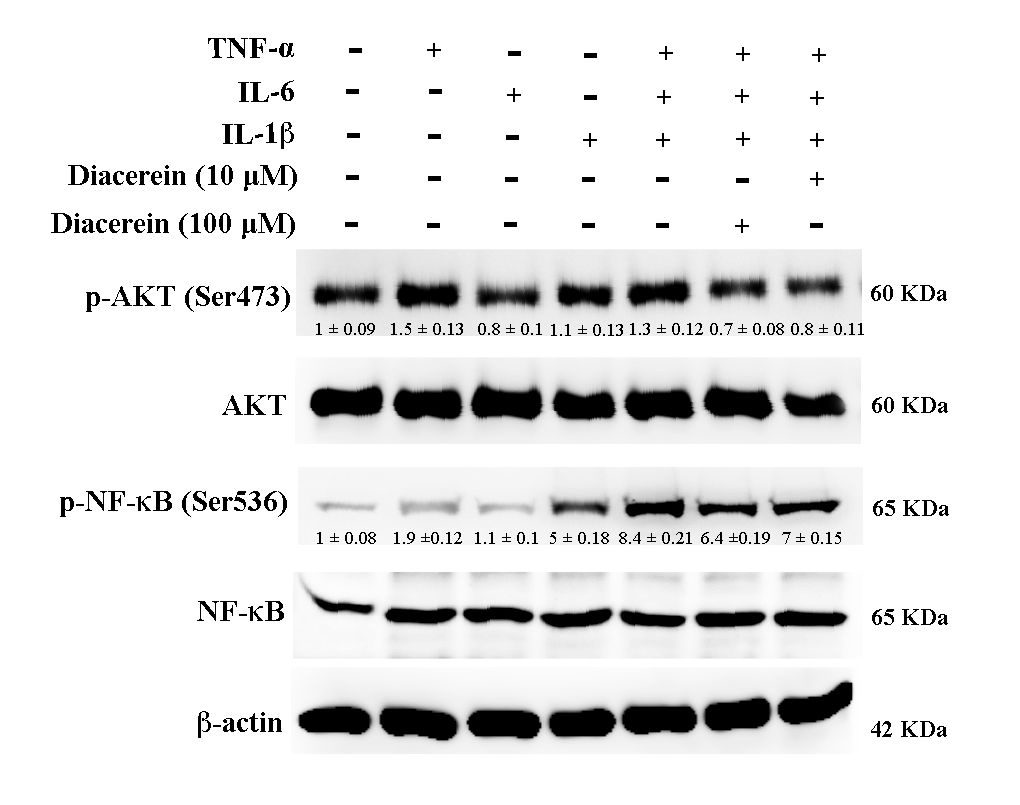
**

**Uncropped western blot images**

p-AKT (Ser473)


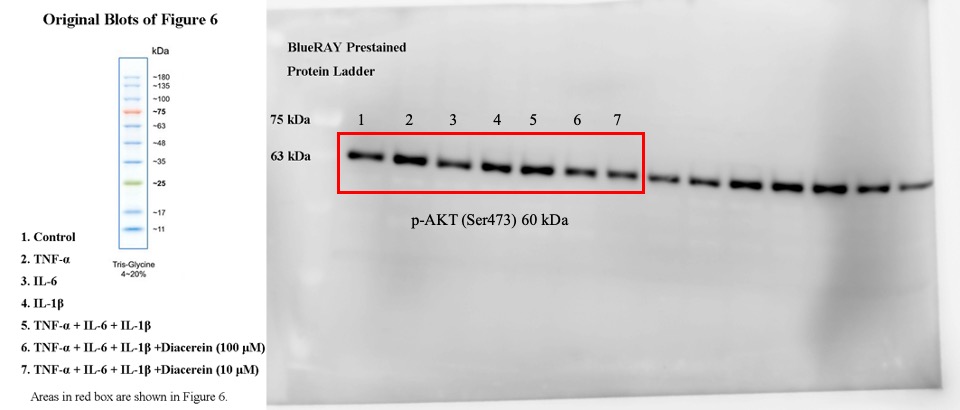


AKT


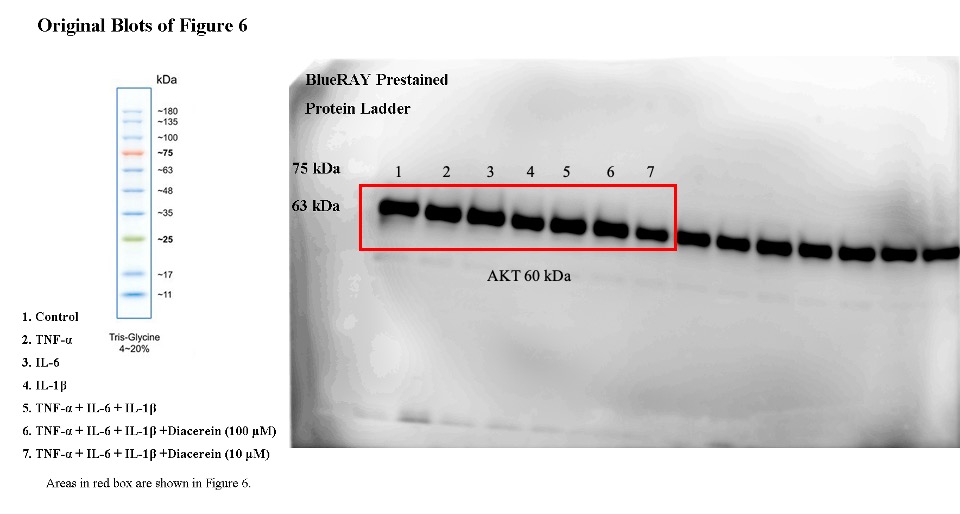


p-NFκB


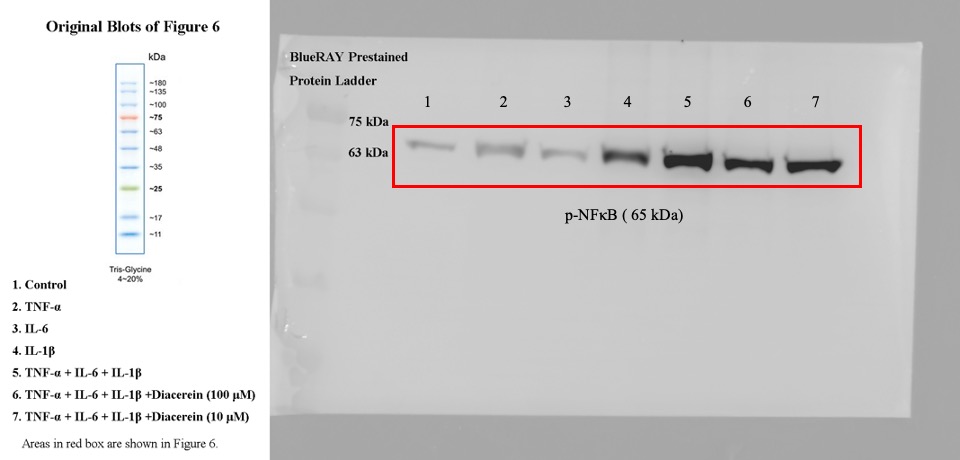


NFκB


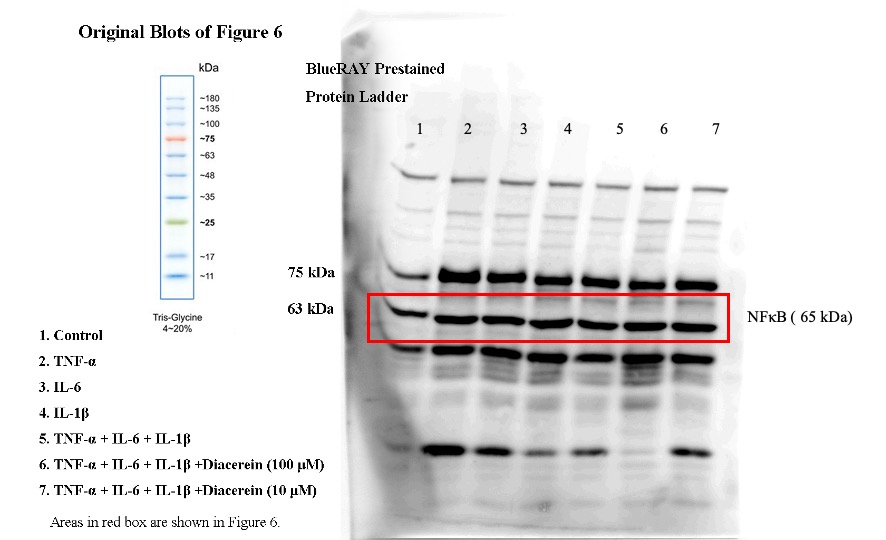


β-actin


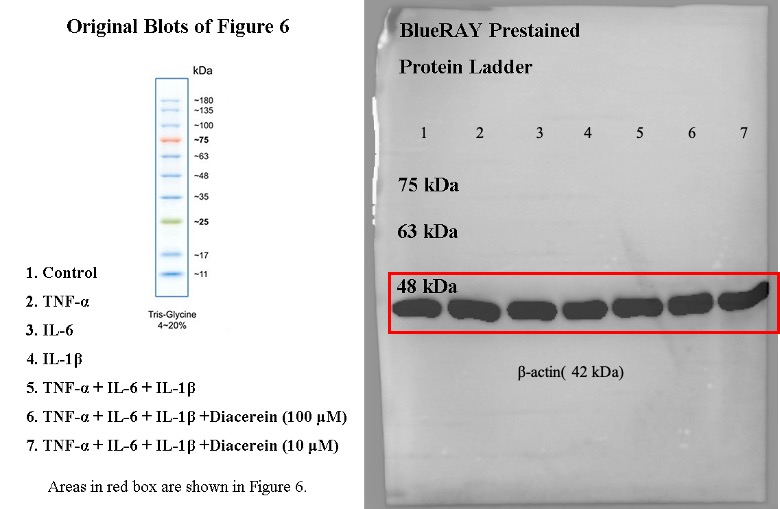


**Supplementary Figure 3:**


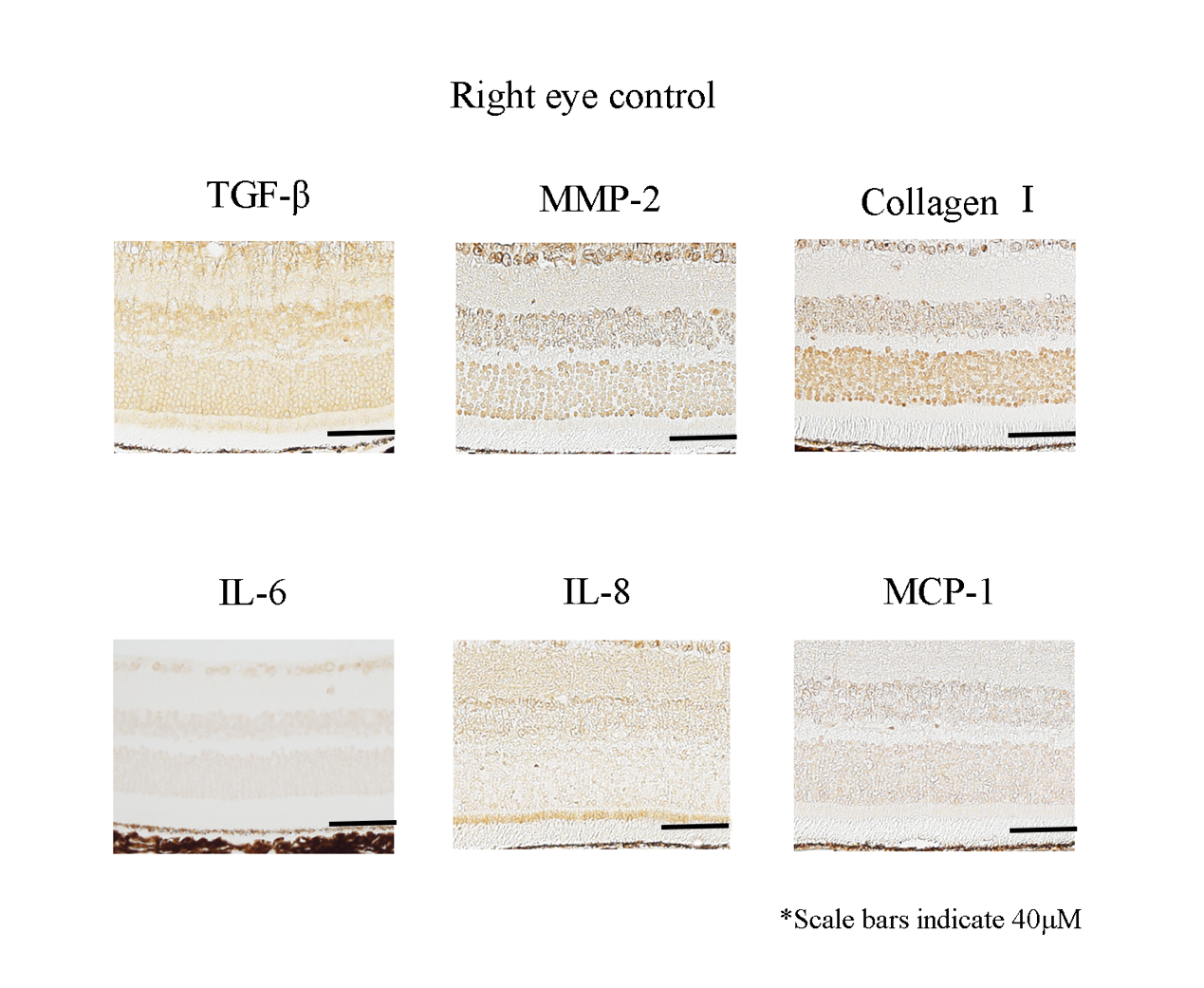


**Supplementary Figure 3: Immunohistochemical analysis of TGF-β1, MMP-2, type Ⅰ collagen, IL-6, IL-8, and MCP-1 expression in Right eye control of a normal hamster.**

**Supplementary Figure 4:**


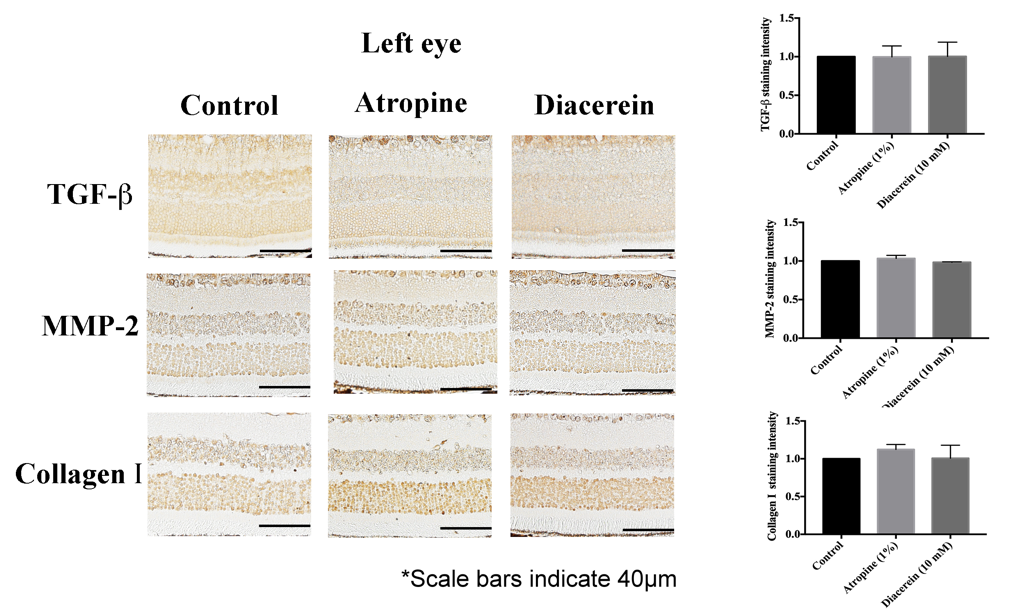


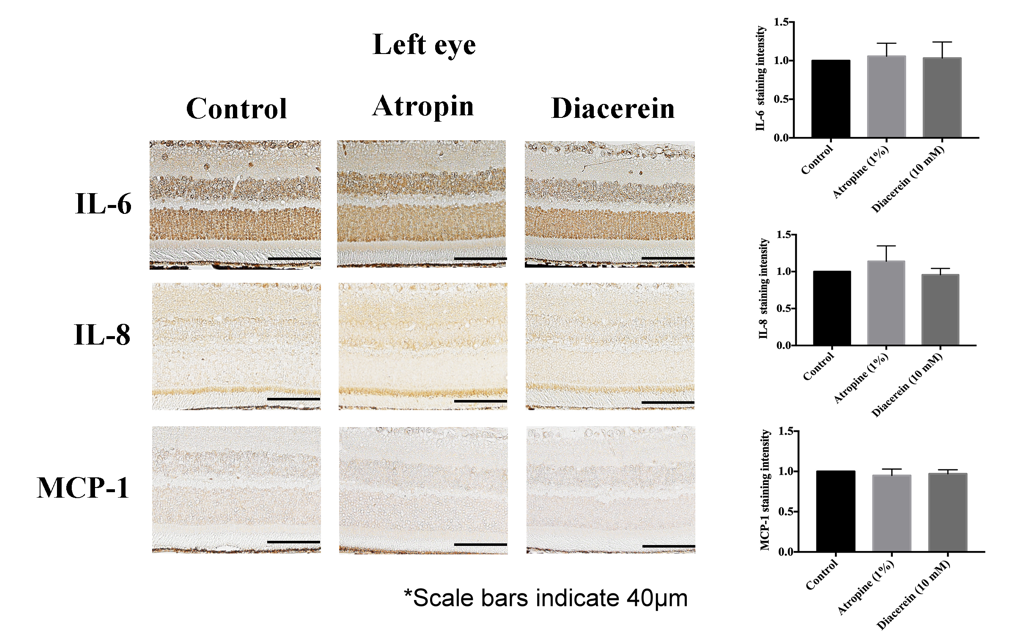


**Supplementary Figure 4: Immunohistochemical and analysis of TGF-β1, MMP-2, type Ⅰ collagen, IL-6, IL-8, and MCP-1 expression in Left eye control, 1% atropine, and 10 mM diacerein of hamster.**
